# A Methodological Framework for Evaluating the Evidence for Complementary and Alternative Medicine (CAM) for Cancer

**DOI:** 10.3390/cancers3010773

**Published:** 2011-02-23

**Authors:** Robert Zachariae, Helle Johannessen

**Affiliations:** 1 Psychooncology Research Unit, Department of Oncology, Aarhus University Hospital, and Department of Psychology, University of Aarhus, Jens Chr. Skous Vej 4, 8000 Aarhus C, Denmark; E-Mail: bzach@aarhus.rm.dk; 2 Research Unit Health, Man & Society, Institute of Public Health, University of Southern Denmark, JB Winsløws Vej 9B, 5000 Odense C, Denmark

**Keywords:** complementary and alternative medicine, evaluation, effects, research methodology

## Abstract

In spite of lacking evidence for effects on cancer progression itself, an increasing number of cancer patients use various types of complementary and alternative medicine (CAM). There is disagreement between CAM practitioners, researchers and clinical oncologists, as to how evidence concerning effects of CAM can and should be produced, and how the existing evidence should be interpreted. This represents a considerable challenge for oncologists; both in terms of patient needs for an informed dialogue regarding CAM, and because some types of CAM may interact with standard treatments. There is a need for insight into which kinds of CAM may work, for whom they work, what the possible effects and side-effects are, and in what ways such effects may come about. The present article presents a framework for evaluating effects of CAM by suggesting a taxonomy of different levels of evidence related to different types of research questions and discussing the relevance of different research methodologies for different types of effects.

## 1. Introduction

Complementary and alternative medicine (CAM) represents a considerable challenge for oncologists, both in terms of patient needs for an informed dialogue regarding CAM, because some types of CAM may interact with standard treatments [1], and because excessive use of some types of CAM, e.g., certain herbal remedies and vitamin supplements, may have detrimental health effects [2,3].

A European survey from 2005 revealed that 36% of cancer patients supplement their conventional treatment with various types of CAM, with usage ranging from 15 to 73% in the 14 countries included in the study [4]. Economically, CAM represents a considerable industry with an estimated $34 billion out-of-pocket expenditure in the U.S., but with the majority of CAM types still remaining to be evaluated [5,6]. CAM is thus part of the clinical reality for many cancer patients today, and for this reason alone, research in CAM and CAM use is highly relevant, both from a societal perspective and to clinical practice.

CAM is a highly diverse category of treatments with large between-country differences in types and usage, but is commonly defined as “diagnosis, treatment, and/or prevention which complements mainstream medicine by contributing to a common whole, by satisfying a demand not met by orthodoxy or by diversifying the conceptual frameworks of medicine” [7]. Although countries differ in their traditions for the naming of these forms of medicine, and although variations in the use of the terms alternative, complementary, and integrative medicine often relate to different positions on conventional treatment, in the following we use CAM as a broad generic term covering therapies that are generally not part of conventional oncology treatment.

Although it is highly debated both how evidence concerning effects of CAM can and should be produced, and how the existing evidence should be interpreted, the majority of CAM research conducted so far has focused on documenting possible effects on physical symptoms and/or psychological well-being. The available results are generally inconsistent, and authors of systematic reviews repeatedly argue that the available research is based on samples that are too small and on research having methodological flaws, and that decisive conclusions therefore cannot be supported [8-11]. The methodological issues may to some degree be associated with the ongoing disagreement among researchers as to what the appropriate methodology for CAM research should be. While conventional medical researchers generally agree that CAM should be subjected to the same rigorous standards applied to studies of conventional therapies, e.g., randomized and placebo controlled trials, CAM practitioners often raise objections to the relevance of conventional scientific methodologies, claiming that these methods do not capture the true effects of CAM. To many practitioners and users of CAM, the subjective experience and report of patients confirming that they experience reduction of symptoms, increased well-being, or simply that they subjectively feel the treatment works, are the most important criteria of effect.

Evidence of effect is an inevitable requirement if a therapeutic method is to be integrated in conventional health care. It is therefore relevant to reconsider how to best investigate effects of CAM. There is a need for insight into which kinds of CAM may work, for whom they work, what the effects are, and in what ways the effects come about. In this paper, we propose a framework for evaluating and discussing effects of CAM by suggesting a taxonomy of effects, discussing the relevance of different research methodologies for different types of effect, and pointing to needs for future research.

## 2. A Framework for Evaluating Effects

When looking at the available clinical research on CAM, it is obvious that the qualities usually adhered to in clinical trials are only partially met. Usually, studies of CAM for cancer are rather small, and a number of aspects such as blinding and randomization are often lacking, although some improvement has been seen within the last decade. For example, a brief overview of clinical studies of massage administered to cancer patients reveals that the six identified studies published from 1985–1999 included an average of 36 patients and only four studies had control groups (cross-over design, massage without essential oils, or rest). During the period 2000–2007, a further 18 studies were published, with a mean of 65 patients and all with control groups (standard care, conversation, or placebo treatment). In 2008, three new randomized controlled trial (RCT)s investigating massage for cancer were published with a mean of 214 patients in each study and randomization of patients to massage or control, but still lacking information on whether patients, practitioners or data-processing personnel were blinded [12]. No multi-center studies or meta-analyses have been identified, and one recent review states that it was not possible to conduct a meta-analysis of studies of massage in the care for patients with cancer due to the wide range of massage techniques, therapeutic settings, and patient profiles [13]. As demonstrated by this brief sketch, while the number of studies on massage for cancer patients per year as well as the number of patients included in the studies is slowly increasing, as is concurrency with standard demands of RCTs, there is still a way to go before research on massage to cancer patients resembles ongoing research of conventional treatment effects. The same appears to be the case for many other types of CAM.

The size of a study sample, inclusion of control groups, and blinding are formal design issues. Another issue concerns what research questions a study actually addresses. In recent years, it has been a requirement in oncology research to test a new therapy not only for its ability to cure the cancer, but also to assess its possible side effects and the implications for the quality of patients' lives. Addressing all of these issues in clinical trials provides practitioners and patients with an improved basis for informed choice of treatment. One could argue that if oncologists should be able to discuss the use of CAM with their patients, the same issues should preferably be covered in CAM research. However, the discussions may need to be framed differently, as no form of CAM so far has been shown to influence the prognosis of cancer in large and well conducted RCTs. Patients, nevertheless, often wish to discuss their use of CAM with the oncologist, and oncologists need a suitable framework for discussing the available evidence or lack of evidence with respect to CAM.

We suggest that when exploring the level of evidence for the effects of a treatment, a full investigation must include information on (I) subjective, self-reported effects, e.g., effects on nausea, pain, or general well-being; (II) objective, physiological effects; e.g., changes in tumor size or relevant immune and endocrine parameters, and (III) whether the mechanisms of effect correspond to the theory of the particular treatment, e.g., evidence that water molecules can retain the “memory” of a given substance as proposed in homeopathy. These three elements cover different, complementary aspects of the question concerning therapeutic effect, and one can argue that all three should be well documented before the efficacy of a given treatment can be considered to be well established. As an aid to more productive discussions on effects of CAM, we propose a clearer distinction between different explanatory levels of evidence (Table 1).

At evidence level A, we would find studies that are able to establish and confirm all three aspects of effects of the tested therapy. Evidence for the effects of a treatment at level A requires evidence of significant improvement of specific patient-reported symptoms as well as improvements in specific physiological symptoms compared to placebo, or if an effective standard treatment is available, treatment as usual. Furthermore, level A requires that mediating mechanisms that correspond to the mechanisms proposed by the theory of the treatment in question have been tested and verified.

At level B, we find studies that have provided evidence of improvement of specific patient-reported symptoms as well as improvement of specific, relevant physiological processes, when compared to treatment as usual or placebo, but no evidence of, or perhaps even evidence contrary to, the mechanisms proposed by the theory of the treatment.

Evidence level C refers to treatments for which there is evidence of significant improvement of specific subjective symptoms as well as relevant physiological measures, but where the effects do not differ from those obtained by placebo and therefore may be attributed to “non-specific” psycho-physiological mechanisms.

At evidence level D and E, we find studies with results that indicate self-reported effects, but where it has neither been possible to document effects on any physiological symptoms nor to document the mechanisms of effect. In the category D, the treatment outcomes are specific (e.g., fatigue, nausea, anxiety), while they are general or non-specific in category E (e.g., general well-being). Finally, level F refers to the situation where it has neither been possible to document any significant effect on self-reported well-being nor on specific symptoms, and for which there are no documented mechanisms of effects.

We believe that the taxonomy outlined in Table 1 could be useful in classifying the available evidence for various therapeutic interventions. Today, biomedical cancer treatments are tested not only for their physiological effects but also with respect to subjective effects (e.g., side effects and other aspects of quality-of-life), and the working mechanisms are generally well known. While a substantial number of conventional cancer therapies in use today are well-documented at evidence level A, we have been unable to identify any CAM modality for which there is the same level of documentation. While this may be the reason why they are categorized as complementary and alternative, it does not necessarily mean that they are of no value. There may thus be treatments that have no documented biological effects on cancer, and for which we do not know the working mechanisms, but nevertheless may help cancer patients feel better and improve their quality-of-life. The issue is not simply a question of whether there is evidence or not, but of what type of evidence.

## 3. Research Questions and Methodological Challenges

The overall framework presented in Table 1 can be further elaborated into specific types of research questions and their corresponding methodologies. We suggest that research questions and hypotheses can be structured according to at least six domains of questions relating to the three main aspects of evidence proposed in Table 1. We also suggest that the research questions asked may require different methodologies to be answered in a satisfactory manner, given that various methods have their strengths and weaknesses within each of the different domains. The research questions that can be asked, relevant research designs, and examples of methodological challenges within each of these domains are summarized in Table 2. As a more detailed illustration of the model, we will examine a few examples of CAM research at each level.

### 3.1. Testing Theories of Mediating Mechanisms

To evaluate the evidence at level A, we need to ask the question whether a given effect of a specific therapy is produced by the mechanisms proposed by the theory associated with the therapy in question.

One illustrative example could be so-called *spiritual*- or *energy healing*. While there are several types of energy healing, proponents generally attribute effects of these types of treatment to various sources, e.g., “spirits”, God, “universal forces”, or “biological healing energies” residing in the healer or receiver of healing. Proponents of energy healing generally agree that healing can be performed “*at a distance*” without conscious awareness on the part of the recipient, and the recipient can be any living being, *i.e.*, person, animal, plant or other living system [14]. While the suggested sources, e.g., “spirits”, usually are beyond what can be tested scientifically, the theory of energy healing consists of at least two aspects that while being controversial from a conventional scientific viewpoint may be subjected to scientific testing: 1) that healing can influence any living system, including individual cells, and 2) that the healing can be performed “at a distance” without conscious awareness of the recipient. However, only few published studies have directly attempted to address these questions. Some researchers have investigated whether energy healing can inhibit the viability and growth of cancer cells *in vitro*, some reporting positive results [15,16], while others have been unable to replicate these findings [17]. If well-controlled future studies are able to confirm that cancer cells *in vitro* can indeed be influenced by spiritual healing, this could be taken as supporting both aspects of the theory.

Intercessory prayer, *i.e.*, having a group of individuals pray for a group of patients without the patients being aware that they are being prayed for, is another example of spiritual- or energy healing. A small but growing number of randomized clinical trials have investigated whether this form of intervention may have a positive influence on patients' health status [18]. Most studies have focused on other diseases than cancer, e.g., coronary heart disease, and while there are positive reports [19], the findings have generally been negative [20]. The methodological quality and the interpretability of the available results is limited, but, again, if results of future well-controlled studies consistently confirm effects of intercessory prayer that cannot be explained by other factors, this could be taken as supporting at least the theoretical claim that energy healing works at a distance.

Several other widely used types of CAM involve theories of working mechanisms that are controversial within conventional science. One example is acupuncture, which is often used by cancer patients to reduce treatment-induced side effects, e.g., nausea. While there are variations, most acupuncture treatments are based on ancient Chinese theories claiming that the health of an organism depends on the flow of the “life force” of *Qi*, and that disease stems from imbalances and blocking of the flow of *Qi* through *meridians* in the body [21]. The traditional Chinese theory of acupuncture implies that bodily balance can be restored by stimulating specific meridian points that are believed to correspond to specific clusters of organs, sentiments, and qualities, e.g., temperatures of hot or cold [22]. There have been several attempts to document the existence of the meridians. Photographic images or measurements of electric currents supposedly revealing meridians in the body have been provided as proof of their existence, but the validity of these techniques has been questioned by other researchers, and the existence of meridians is still undocumented scientifically [23]. From the theoretical viewpoint, a fundamental question concerning the validity of the traditional theories of acupuncture is whether acupuncture needles must be inserted in specific points to have an effect. While adherents of the theory insist that individual specific points have identifiable and reproducible clinical effects, some practitioners of modern (Western) medical acupuncture find that it makes little difference exactly where the needles are inserted [24]. This conclusion is supported by results from several large multi-center trials showing that while acupuncture may be more effective than no treatment, it is generally not better than sham acupuncture in non-specific points [25-27]. In addition, such findings do not rule out the possibility that the effects of acupuncture are due to psychological mechanisms, e.g., “placebo-effects” resulting from positive expectation on the part of the patient and/or the practitioner. Various forms of “placebo-acupuncture” have been suggested, including placebo-needles that are claimed to enable double-blinding but do not penetrate the skin as well as other forms of so-called minimal acupuncture using blunt needles or superficial needling [28], although there is still some disagreement concerning the validity of these methods [29]. Other approaches involve using brain-imaging techniques to compare results of stimulating specific points to those obtained by sham acupuncture. While there have been some reports of point specificity, e.g., increased blood flow in the auditory cortex following stimulation of an ear-specific point, others have been unable to replicate these findings [30].

Homeopathy is another form of therapy that has become increasingly popular among cancer patients [4], and some cancer patients have even been reported to use homeopathy as an alternative to conventional treatment, with increased recurrence and mortality as a result [31]. Like energy healing and classical acupuncture, homeopathy is based on theoretical mechanisms that differ markedly from anything known within conventional treatment. The fundamental theorem of homeopathy, ‘similia similibus curentur’ (like cures like), was formulated by the founder of homeopathy, the German medic Samuel Hahnemann (1755–1845), and refers to a working principle stating that the symptoms provoked by a substance in its natural form will be cured by the same substance highly diluted [32]. The production of homeopathy involves repeated dilutions and shaking (probing) of the original substances, sometimes to the extent that the chance to find any of the original substance is close to null. In theory, the higher the dilution, the stronger the effect. The therapeutic effect has been explained to result from a type of “memory” of the original substance retained in the water molecules of the dilution [33]. An attempt to test this hypothesis is represented by Jacques Beneviste, who in 1988 published a controversial paper in *Nature* reporting effects of highly diluted anti-IgE on degranulation of basophil cells. The paper was later withdrawn, when attempts to replicate the findings failed [34], and the theory behind homeopathy still remains invalidated [35].

As demonstrated above, the validity of theories of working principles of many complementary therapies have not been supported by research, and research designs specifically tailored to test the theories in question are needed. To provide evidence at level A for a given CAM, there is a need for developing designs that both meet conventional scientific requirements *and* are able to test the theory of the CAM in question on “its own terms”. While this may be technically possible in some instances, other CAM theories may be unsuited for scientific testing, *i.e.*, by referring to immeasurable entities.

### 3.2. Specific and General Physiological Effects

Evidence at levels B and C are concerned with physiological effects. While it may not be possible to find evidence for the theory of a therapy or even to test it at the theoretical level, e.g., the failure to validate the theory of point specificity in acupuncture, this does not exclude the possibility that the therapy has identifiable effects at the specific physiological level. At the level of physiological effects, the key question is whether some forms of CAM are able to produce relevant physiological effects, e.g., reduce cancer growth, improve relevant immune measures, reduce observable side-effects, or prolong survival.

It has been demonstrated in several studies that the use of complementary medical products is by far the most popular supplement to conventional cancer treatment among cancer patients. Shark cartilage, for instance, has for some decades been popular among cancer patients. The basis for this popularity has been the claim that sharks rarely get cancer because of the high proportion of cartilage in their bodies, and some laboratory research has suggested the existence of antiangiogenic and anti-tumoral compounds in shark cartilage [36]. However, sharks do in fact develop cancer [37], and the results of randomized clinical trials with cancer patients have been disappointing [38]. Another example is mistletoe extract, which is often used by cancer patients and is claimed to improve survival. However, a recent meta-analysis of the available randomized clinical trials has revealed no effect on survival [39].

When testing potential specific physiological effects of a CAM, e.g., on tumor growth, survival or side-effects, disregarding whether the treatment works according to its own theoretical claims or not, the golden standard of clinical research, *i.e.*, the RCT, remains the ideal methodological choice. When testing herbal medicines or other non-conventional medicines, e.g., shark cartilage, mistletoe or antioxidants, it should be possible to evaluate the evidence at level B. Here RCTs do not pose any serious methodological problems, as the proposed active substance usually can be tested in a double-blinded manner against an inactive substance—usually in addition to treatment-as-usual. It should be noted, however, that research in other areas suggests that since active substances are often associated with side-effects that are not found in the usual inactive placebo-treatments, this may threaten the efficacy of a blinding procedure, thereby increasing the risk of type-1 error [40].

As suggested above, other unconventional therapies pose more serious methodological challenges. The difficulties in providing acceptably valid double-blinded conditions for acupuncture remains a serious hindrance for the testing of whether this type of CAM has any relevant clinical effects beyond placebo, *i.e.*, at evidence level B. The same applies to other types of CAM, such as Qigong, a Chinese type of meditative exercise. Although there are reports of improved survival and reduced tumor size among cancer patients practicing Qigong, the methodological quality of the available studies is limited, and so far, no double-blinded clinical trials have been conducted [41], and hence claims for clinical efficacy can be questioned. Here it should, however, be noted that blinding is also often not possible or ethical when testing conventional cancer treatments, e.g., chemo- or radiotherapy, due to the nature of these treatments.

### 3.3. Self-Reported Effects on Symptoms and Well-Being

Evidence levels D and E are concerned with self-reported effects. Besides physiological aspects observable by a third party, e.g., the size of a tumor, a number of physical consequences of cancer and cancer treatment, including nausea, fatigue, and pain, can only be assessed by patient self-report. Nausea as a common side effect of chemotherapy and the experience of pain as an adjunct symptom connected with cancer, are highly discomforting for cancer patients, and it could therefore be worthwhile to look for CAM as a potential remedy for such symptoms and problems. Meta-analyses of the available literature indicates, for instance, that acupuncture may reduce nausea after chemotherapy, although the mechanisms are still unknown [42]. The research also suggests that acupuncture may have analgesic effects, even though such effects can be found for any acupuncture point and not only for theoretically specific points [24].

Questionnaire-based investigations of health-related quality-of-life represent another approach to evaluating self-reported effects of CAM, and the literature indicates the possibility of some effects in this area. For instance, although mistletoe therapy has not been shown to prolong survival time, there is some evidence to suggest that it may provide some improvement of quality-of-life for cancer patients [39]. Our data from an ongoing nationwide cohort study of Danish women treated for early stage breast cancer [43] indicate that almost 40% of the women had used some type of CAM within 3–4 months after surgery, and more than 50% had used CAM within the following 12 months. While approximately one-third of the CAM users in this cohort were either absolutely or relatively certain that their CAM would have a beneficial effect on the disease itself and 39% believed an effect was possible, the remaining 27% did not believe that the CAM they was using would have any effect on the disease, suggesting that for many patients, cure or prolonged survival may not, as discussed below, be the primary reason for use [44].

### 3.4. Treatment as a Meaning-Generating Activity

As indicated above, although there is no clear evidence of relevant physiological effects, quantitative questionnaire-based studies suggest that the use of CAM may be associated with self-reported effects on various aspects of quality-of-life of cancer patients. These findings may be complemented by studies based on qualitative methodologies, e.g., interviews and participant-observation. A growing number of studies have applied qualitative methods to the investigation of patients' experience of effects of CAM-modalities, and although the number of participants in these studies is usually small, they may provide substantial clues as to why cancer patients use CAM, thereby providing explanations for the continued use in spite of the lack of hard evidence of effects. The available qualitative studies have rarely been concerned with patients with one type of cancer and generally attempt to explore cancer patients' subjective experiences of living with a malignant disease, and the results indicate that CAM may be a tool for adapting to the new life situation of living with cancer.

In an interview study at a clinic of integrative medicine in Vancouver [45], the stories of 11 participants suggested that they underwent a transformation process involving learning about themselves, of becoming more aware of who they are and how they relate to the world. This new understanding about their selves and how they related to others seemed to provide participants with a new direction for improving their lives notwithstanding the cancer they suffered. The analysis suggested that this process of personal development evolved along a process of four stages: looking to options other than conventional treatment; doing ‘inner work’, *i.e.*, revisions of conceptions of what life and illness is all about; witnessing a shift in inner well-being, *i.e.*, a redirection of goals and means in life; and seeing the world and oneself through new eyes. Similar aspects associated with the use of CAM were found in an interview study comparing breast cancer patients using anthroposophical medicine and care with matched breast cancer patients using only conventional medicine [46]. The results suggested that the women using anthroposophical medicine, to a greater extent than those using only conventional medicine, emphasized the importance of self-reflection and the meaning-of-life. Important changes noticed were an appreciation of the beauty of life, experiences of threat, introspection into self and meaning of life, and changes in the body. The group that received anthroposophical care seemed to be more orientated towards personal growth and meaning of life, whereas the matching group was more orientated towards external activities and bodily changes. Important issues were the women's openness to their own vulnerability, combined with their search for emotional expansion and the necessary strength to meet this vulnerability. The study points to the guidance towards meaning, purpose in life, and increased quality-of-life as potential implications of CAM use. In a third study, Billhult and Dahlberg interviewed eight cancer patients receiving massage as an adjunct therapeutic intervention. This study identified five themes of importance to the participants: the massage was meaningful because (1) it offered the patient an experience of being “special”; (2) contributed to the development of a positive relationship with the staff, (3) supported a subjective feeling of being strong, and (4) supported a balance between autonomy and dependence. Finally, (5) the massage supported an immediate experience of “feeling good” [47].

That CAM may provide meaningful options for cancer patients is further supported by our research of Italian cancer patients suggesting that the use of CAM may provide patients with means to act in a meaningful way when facing cancer [48]. In one example, a patient suffering from cancer of the colon with metastases to the liver reported use of several forms of herbal medication. In repeated interviews, the patient never mentioned the herbal medicines as curative treatment, as he clearly considered that to be taken care of at the hospital. Instead he emphasized the need to stimulate the organs that were hampered by the disease, especially since they were important organs for the uptake of nutrients, and found it very important to feed the body well when suffering from cancer and undergoing the harsh conventional treatments. This man's story is typical in the sense that it reflects a distinction between curative cancer treatment provided at the hospital, and complementary medicines employed to cope with side effects of hospital treatment and to support the functioning of diseased organs. Italian patients that used herbal medicine and homeopathy generally expressed this distinction in expectations to different forms of medicine, and users of CAM generally reported themselves to be less burdened with side-effects of chemotherapy, when comparing themselves to those patients that did not use complementary medicines [49].

The studies using qualitative methods generally include small numbers of informants, and only few include control groups, which limits the generalizability of results from each study on its own. Taken together, however, all such studies point to subjective experiences of CAM as an important factor in the creation of meaning in the midst of the chaotic and frightening experience of a life-threatening disease. We cannot know, among other things due to the lack of longitudinal designs and control groups, whether the use of CAM is the product of or the reason for the meaning-creating processes observed. Although there is no evidence to suggest that CAM may cure cancer or improve cancer-related physical outcomes, the limited evidence suggests that CAM use may have the potential to be experienced as purposeful by some patients, and may thus help these patients cope with their cancer. Further studies with larger, representative samples are needed to explore the generalizability of these findings.

## 4. Conclusions

There is no doubt that use of CAM is frequent among cancer patients, and many seek CAM as a means to combat the disease. This aim is understandable, considering the seriousness of the disease and the limited effectiveness of many conventional cancer treatments. However, in spite of decades of research on CAM modalities, the available results have been unable to verify the suggested working mechanisms of several widely used types of CAM, and for energy healing, acupuncture, homeopathy, and many other forms of CAM, there is no evidence at level A.

Many patients turn to CAM to find some alternative or supplementary form of cure or life-prolonging effect, but while there have been some reports of promising results, none of the available systematic reviews, including meta-analyses, have been able to support claims of CAM with respect to curing or improving the prognosis of cancer. It is, however, important to note that since double-blinding is often difficult or not possible due to the character of many forms of CAM (e.g., acupuncture and physical exercises like Qigong, *etc.*), it may be necessary to accept RCTs without blinding of the patients or practitioners as the best possible evidence that can be obtained. However, it should be noted that this is also the case in trials of many conventional cancer treatments, e.g., radio- and chemotherapy. Regardless of the validity of the proposed mechanisms, the evidence of effects on cancer-relevant physical health outcomes at level B is limited.

Taken together, documented effectiveness of CAM seems primarily to be found for issues related to self-reported effects. Questionnaire-based investigations suggest that patients may choose to use CAM for reasons other than cure or improvement of survival, and results from clinical trials suggest the possibility of reductions in self-reported nausea and pain after acupuncture, or increases in measures of quality-of-life after treatment with mistletoe. While documented physiological effects associated with the self-reported outcomes are rare (level C), the available findings suggest that finding evidence at level D and E is most likely for selected forms of CAM.

Finally, results from qualitative studies indicate that CAM may be a useful tool for the creation of meaning in a life situation that involves considerable distress for many patients. While many of the available qualitative studies, due to methodological limitations such as lack of control groups, are unable to establish whether CAM is the cause for the creation of meaning reported by patients, their results nevertheless suggest the potential usefulness of CAM in the search for subjective experiences of meaning and existential relief for those patients who choose to use CAM.

For future research on CAM for cancer we see a number of challenges that must be dealt with. The first is to create research designs that are able to include a range of assessment tools in the same study. Research could move one step forward by employing several approaches in one trial; *i.e.*, simultaneous investigations of whether a given therapy has any impact on physiological measures, on self-reported measures, and/or with respect to establishing meaning or existential relief to the patient in a situation characterized by fear and uncertainty. Cross-disciplinary studies of different CAM modalities may provide a clearer picture of what CAM may and may not provide for cancer patients than the single-disciplinary studies available today. Furthermore, the issue of blinding has to be solved with respect to many forms of CAM—perhaps by renouncing on the common demand for double- or triple-blind trial designs. Finally, it may be necessary to renounce on the demand of randomization with regard to trials of CAM that involve personal involvement of the patients given that such forms of therapy may require a certain willingness to collaborate on behalf of the patients. This could be the case in therapies that are based on repeated physical exercises (e.g., qigong) or are contrary to everyday experiences (e.g., spiritual healing for persons living in secular societies). Regardless of whether randomization is involved or not, control groups of patients receiving sham treatment or treatment-as-usual are possible to establish and should be employed to distinguish between the effectiveness of the therapy of the trial and the natural development of disease and recovery. If it turns out that it is possible to provide documentation of effect at any of the proposed levels, it would be relevant to establish experiments that could shed light on the working mechanisms of the form of therapy involved.

If used when designing and reporting results of individual studies as well as systematic reviews of the available evidence, the proposed taxonomy could provide the oncologist with a tool to assist cancer patients in making more well-informed choices concerning use of and expectations to CAM.

## Figures and Tables

**Table 1.** Explanatory levels of evidence.

**Explanatory Level of Evidence**	**Documented Subjective Effect**	**Documented Physiological Effect**	**Documented Mechanisms Corresponding to the Theory of the Treatment**
	**I**	**II**	**III**
A^1^	+	+	+
B^2^	+	+	-
C^3^	+	+	-
D^4^	+	-	-
E^5^	+	-	-
F^6^	-	-	-

1 Evidence of specific effects beyond placebo and evidence supporting the proposed theory of treatment.

2 Evidence beyond placebo of effects on specific symptoms.

3 Evidence of effects on specific symptoms, but no difference from placebo, indicating non-specific mechanisms.

4 Evidence of self-reported effects on specific symptoms, but no documented physiological effects.

5 Evidence of effects on self-reported general well-being, but no documented physiological effects.

6 No evidence of any self-reported or physiological effects.

**Table 2.** Relevant research questions, designs and methodological challenges.

**Research Domain**	**Explanatory Level of Evidence**	**Research Question**	**Relevant Designs**	**Examples of Methodological Challenges**
I. Theoretical	A	Does the therapy have subjective and physiological effects that can be explained by the mechanisms proposed by its theory	Controlled experiments investigating the proposed mediating mechanisms	Developing research designs capable of testing the theory on its own premises, while controlling for possible alternative explanations.
II. Specific physiological	B	Does the therapy have documented specific physiological effects beyond those than can be attributed to expectancy and other non-specific mechanisms (placebo)?	Double-blinded, randomized, placebo-controlled trials	Choosing relevant and valid physiological measures, ensuring effective blinding of conditions, controlling for natural improvement
III. Non-specific physiological	C	Does the therapy have documented physiological effects, regardless of the mechanisms involved?	Randomized, controlled trials	Choosing relevant health-related physiological measures and controlling for natural improvement
IV. Specific self-reported	D	Does the therapy have documented specific self-reported effects beyond those than can be attributed to expectancy and other non-specific mechanisms (placebo)?	Double-blinded, randomized, placebo-controlled trials	Choosing valid, specific self-report measures. Controlling for non-specific factors and natural improvement
V. Nonspecific self-reported	E	Does the therapy have non-specific effects on various aspects of well-being?	Randomized, controlled trials	Choosing relevant and valid self-report measures, controlling for non-specific factors and natural improvement
VI. Individual meaning	E	What are the patients' experiences of the therapy and its effects?	Studies of patients' health-beliefs, expectations, and perceptions of the therapy and its effects.	Adequate study size, transparency and validity in research methodology, comparisons with non-users.
